# Rational confederation of genes and diseases: NGS interpretation via GeneCards, MalaCards and VarElect

**DOI:** 10.1186/s12938-017-0359-2

**Published:** 2017-08-18

**Authors:** Noa Rappaport, Simon Fishilevich, Ron Nudel, Michal Twik, Frida Belinky, Inbar Plaschkes, Tsippi Iny Stein, Dana Cohen, Danit Oz-Levi, Marilyn Safran, Doron Lancet

**Affiliations:** 1 Department of Molecular Genetics, Weizmann Institute of Science, Rehovot, Israel; 2 National Center for Biotechnology Information, Present Address: National Institutes of Health, Bethesda, MD USA; 3 Present Address: Institute for Systems Biology, Seattle, WA USA

## Abstract

**Background:**

A key challenge in the realm of human disease research is next generation sequencing (NGS) interpretation, whereby identified filtered variant-harboring genes are associated with a patient’s disease phenotypes. This necessitates bioinformatics tools linked to comprehensive knowledgebases. The GeneCards suite databases, which include GeneCards (human genes), MalaCards (human diseases) and PathCards (human pathways) together with additional tools, are presented with the focus on MalaCards utility for NGS interpretation as well as for large scale bioinformatic analyses.

**Results:**

VarElect, our NGS interpretation tool, leverages the broad information in the GeneCards suite databases. MalaCards algorithms unify disease-related terms and annotations from 69 sources. Further, MalaCards defines hierarchical relatedness—aliases, disease families, a related diseases network, categories and ontological classifications. GeneCards and MalaCards delineate and share a multi-tiered, scored gene-disease network, with stringency levels, including the definition of elite status—high quality gene-disease pairs, coming from manually curated trustworthy sources, that includes 4500 genes for 8000 diseases. This unique resource is key to NGS interpretation by VarElect. VarElect, a comprehensive search tool that helps infer both direct and indirect links between genes and user-supplied disease/phenotype terms, is robustly strengthened by the information found in MalaCards. The indirect mode benefits from GeneCards’ diverse gene-to-gene relationships, including SuperPaths—integrated biological pathways from 12 information sources. We are currently adding an important information layer in the form of “disease SuperPaths”, generated from the gene-disease matrix by an algorithm similar to that previously employed for biological pathway unification. This allows the discovery of novel gene-disease and disease–disease relationships. The advent of whole genome sequencing necessitates capacities to go beyond protein coding genes. GeneCards is highly useful in this respect, as it also addresses 101,976 non-protein-coding RNA genes. In a more recent development, we are currently adding an inclusive map of regulatory elements and their inferred target genes, generated by integration from 4 resources.

**Conclusions:**

MalaCards provides a rich big-data scaffold for in silico biomedical discovery within the gene-disease universe. VarElect, which depends significantly on both GeneCards and MalaCards power, is a potent tool for supporting the interpretation of wet-lab experiments, notably NGS analyses of disease. The GeneCards suite has thus transcended its 2-decade role in biomedical research, maturing into a key player in clinical investigation.

## Background

Human diseases are at the heart of extensive research encompassing genomics, bioinformatics, systems biology, and systems medicine. Advances in the past decade have seen the rise of high-throughput sequencing techniques, which are collectively referred to as next-generation sequencing (NGS). NGS has increased the cost-effectiveness of sequencing per base, which allows for simultaneous examination of multiple genes through one single reaction, having a crucial clinical utility. A key challenge in this realm is the interpretation of NGS results, whereby identified filtered variant-containing genes are to be related to the patient’s disease phenotypes.

Thus, reliable connections between human genes and diseases need to be established. Different methods may identify such associations, including genome-wide association studies (GWAS), classical genetic studies, transcriptomics and proteomics, functional molecular studies and literature text mining [1]. Such heterogeneous datasets should then be cleverly integrated to allow gene prioritization. For this, integrated searchable databases for genes and diseases are crucial. Furthermore, there is a need for heuristics that connect the realm of NGS with such data structures.

The biomedical world is starting to transition from exome sequencing to whole-genome sequencing (WGS) [2], catalysed by the introduction of technologies that make such analysis significantly more affordable. While the promise of this transition is substantial, the relevant bioinformatics analyses pose significant challenges. The main advantages of WGS are: (1) better protein-coding exon coverage, including recently-discovered genes not currently in the exome capture kits; (2) complete coverage of non-coding exons; (3) full coverage of introns; (4) full coverage of promoter regions; (5) much larger coverage of the all-important ncRNAs; (6) a capacity to address the *terra incognita* of the estimated 400,000 enhancers in the human genome; (7) a much stronger capacity to discover and interpret genomic structural (copy number) variations afforded by much more uniform sequence coverage. This spectrum of variants significantly exceeds the standard annotation, variant filtration that is necessary to reduce the number of variant calls for clinical interpretation (e.g. based on population frequency), and phenotype interpretation used for whole-exome sequencing. Therefore, appropriate bioinformatics pipelines should be adopted.

### The GeneCards suite

In the past 2 decades, our group has been developing the GeneCards suite, which includes a set of databases and tools that integrate and utilize information on human genes (GeneCards), diseases (MalaCards) and pathways (PathCards) from 150 sources [3–6]; Table 1. Its main component is GeneCards, a comprehensive web-based compendium of human genes, with numerous annotations in 18 sections, one of which is the disorders section, devoted to diseases associated with the gene. This information is consolidated from 150 data sources and encompassing 147,962 gene entries, including 21,819 protein-coding genes as well as 101,976 non-coding RNA (ncRNA) genes. The GeneCards suite’s disease database is MalaCards [6, 14, 15], which features 19,289 human diseases, with annotations integrated from 69 sources and shown in 15 sections. One of these is the genes section, showing for every disease its related genes. MalaCards effectively addresses some of the major challenges facing disease bioinformatics: disease nomenclature, integration of heterogeneous information from diverse sources, and generation of a comprehensive and consistent view of gene-disease relationships. GeneCards and MalaCards each have behind-the-scene relational tables (a MySQL database) that handle this information, along with a separate index for the search engine.

**Table 1:** The GeneCards suite member databases and tools

Suite member title	Type	Brief description	Relevant publication
GeneCards	Affiliated database	Human gene database	Stelzer et al. (2016) The GeneCards suite: from gene data mining to disease genome sequence analysis, current protocols in bioinformatics [7]
MalaCards	Affiliated database	Human disease database	Rappaport et al. MalaCards: an amalgamated human disease compendium with diverse clinical and genetic annotation and structured search, NAR [6]
PathCards	Affiliated database	Integrated human pathway database	Belinky et al. PathCards: multi-source consolidation of human biological pathways, database [8]
GeneLoc	Affiliated database	Genome locator	Rosen et al. GeneLoc: exon-based integration of human genome maps, bioinformatics [9]
LifeMap	Affiliated database	Embryonic development and stem cell compendium	Buzhor et al. Cell-based therapy approaches: the hope for incurable diseases, future medicine [10]
TGex	NGS analysis tool	Knowledge-driven NGS analysis	Stelzer G. et al. VarElect: the phenotype-based variation prioritizer of the GeneCards suite, BMC genomics [11]
VarElect	NGS analysis tool	NGS phenotyping	Stelzer G. et al. VarElect: the phenotype-based variation prioritizer of the GeneCards suite, BMC genomics [11]
GeneAnalytics	Analysis tool	Gene set analysis	Ben-Ari Fuchs et al. GeneAnalytics: an integrative gene set analysis tool, OMICS [12]
GenesLikeMe	Analysis tool	Related genes finder	Stelzer et al. GeneDecks: paralog hunting and gene-set distillation with GeneCards annotation, OMICS [13]
GeneALaCart	Analysis tool	GeneCards batch queries	Stelzer et al. In-silico human genomics with GeneCards, human genomics [4]

We further portray MalaCard’s utility in both NGS interpretation and in large-scale bioinformatic analyses. We provide an example for deciphering a specific genetic disease using MalaCards, via our VarElect bioinformatic NGS interpretation pipeline, which utilizes several other GeneCards suite tools. The integration of MalaCards information on gene disease associations and on phenotype information within the GeneCards database facilitates the discovery of new connections among biological entities.

## Results and discussion

### The MalaCards disease universe

To help overcome the impediment of disease name unification stemming from source heterogeneity, we obtained 85,000 disease terms from 15 sources that were examined in a predefined order of importance, and used text unification heuristics to define 19,289 main names and their associated 65,000 aliases. The 15 annotative sections for each disease also include disease-related summaries and publications, disease categories and classifications, symptoms, gene variations, drugs, clinical trials, genetic tests and animal models. Several MalaCards sections (summaries, symptoms and aliases) are incorporated into the GeneCards database. This allows the content of such sections to be searchable in the context of a gene-centric database.

**Fig. 1:**
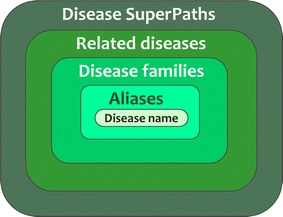
Disease relatedness hierarchy. MalaCards contains several layered levels of relatedness. Aliases are integrated through MalaCards sources via their annotations and our text mining heuristics. Disease families are grouped according to textual similarity, e.g. disease types, inheritance, onset etc. Related diseases are obtained via gene sharing GeneAnalytics [12], a descendent of GeneDecks Set Distiller [13], and by searching within MalaCards. A disease SuperPaths conform to sets of diseases indirectly related to each other via the PathCards algorithm (see “Disease-based pathways”)

Additionally, MalaCards defines a hierarchical disease relatedness scheme, which includes aliases, disease families, related diseases and disease SuperPaths (Fig. 1). SuperPaths are integrated biological pathways from 12 information sources, shown in the PathCards database, a member of the GeneCards suite [14]. This hierarchical disease relatedness scheme constitutes a major MalaCards strength for NGS interpretation, allowing one to gradually augment the sphere of disease definition pertinent to the genes of interest. Currently, the first level (aliases), is available to the GeneCards search index, and therefore also to VarElect (see below). Higher relatedness levels are in the process of being implemented within GeneCards. Further, related diseases form a basis for defining a disease network which we are now using as a platform for disease neighbourhood scrutiny based on MalaCards disease annotations, such as drugs, symptoms and anatomical context.

### The gene-disease network

**Fig. 2:**
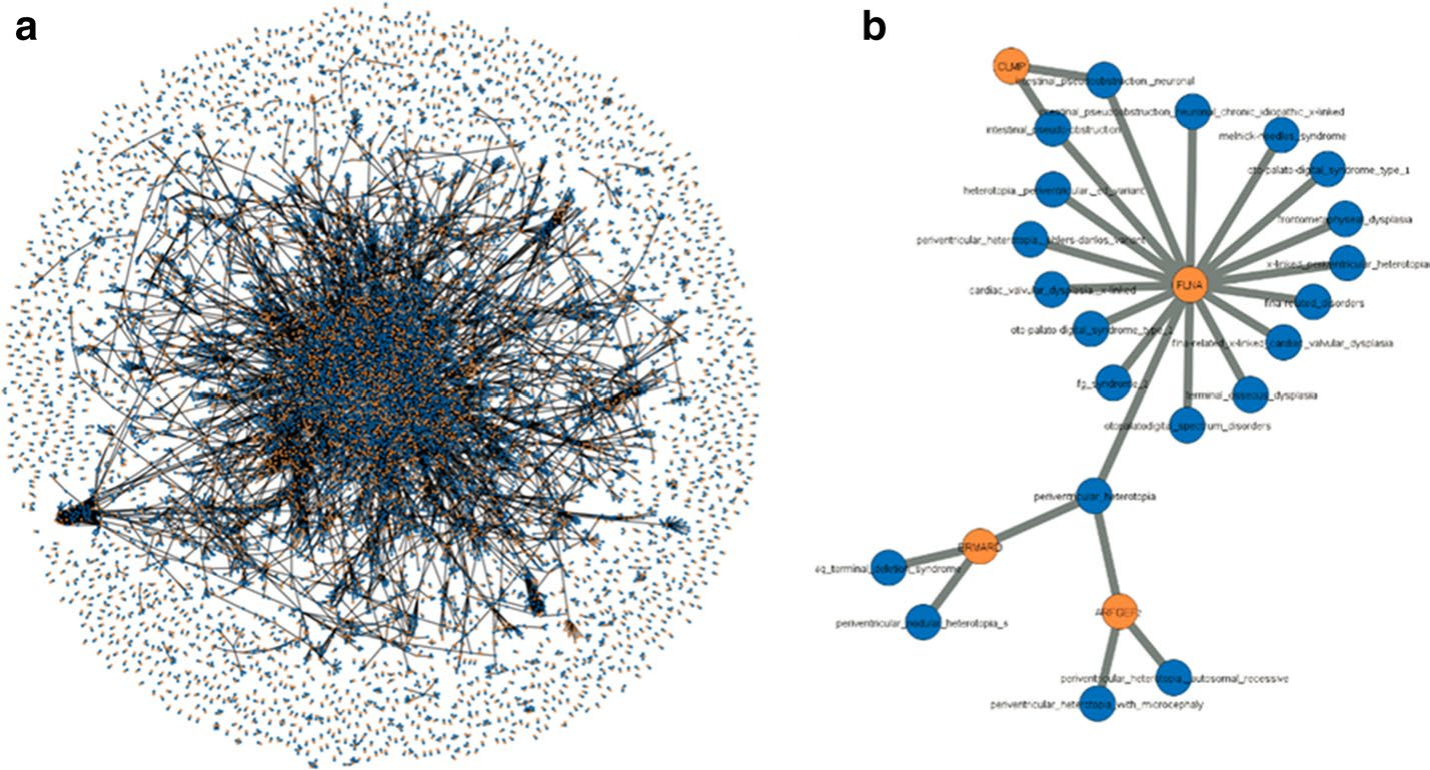
**a** The complete gene-disease network based on MalaCards data. This is a bipartite network with gene and disease nodes, whereby a connection between two diseases is generated via a gene associated with both. The network shown depicts only the more trustworthy disease-gene relationships (“Elite”), as defined in the “Gene-disease network” of the results. **b** One of the connected components (sub-networks) which exemplifies the utility of this network, by demonstrating that MalaCards performs informative grouping of diseases with common symptoms and etiology, which could be useful for off-label therapy or disease mechanism elucidation

Crucial for MalaCards’ NGS interpretation capacities is its well-defined scored gene-disease network. We have generated such a network based on MalaCards data. This network has two types of nodes—genes and diseases, with only one type of edge—that which connects a disease to a gene (Fig. 2). The network is weighted and directional; hence, each pair of nodes is associated with two types of scores: D-G signifies the importance of a gene for the disease, and G-D—the association strength of a disease to the gene. D-G is displayed in the genes section of MalaCards [6, 14] and G-D is portrayed in the disorders section of GeneCards. The latter is also portrayed within the “disease-gene” table affiliated with GeneCards.

**Fig. 3:**
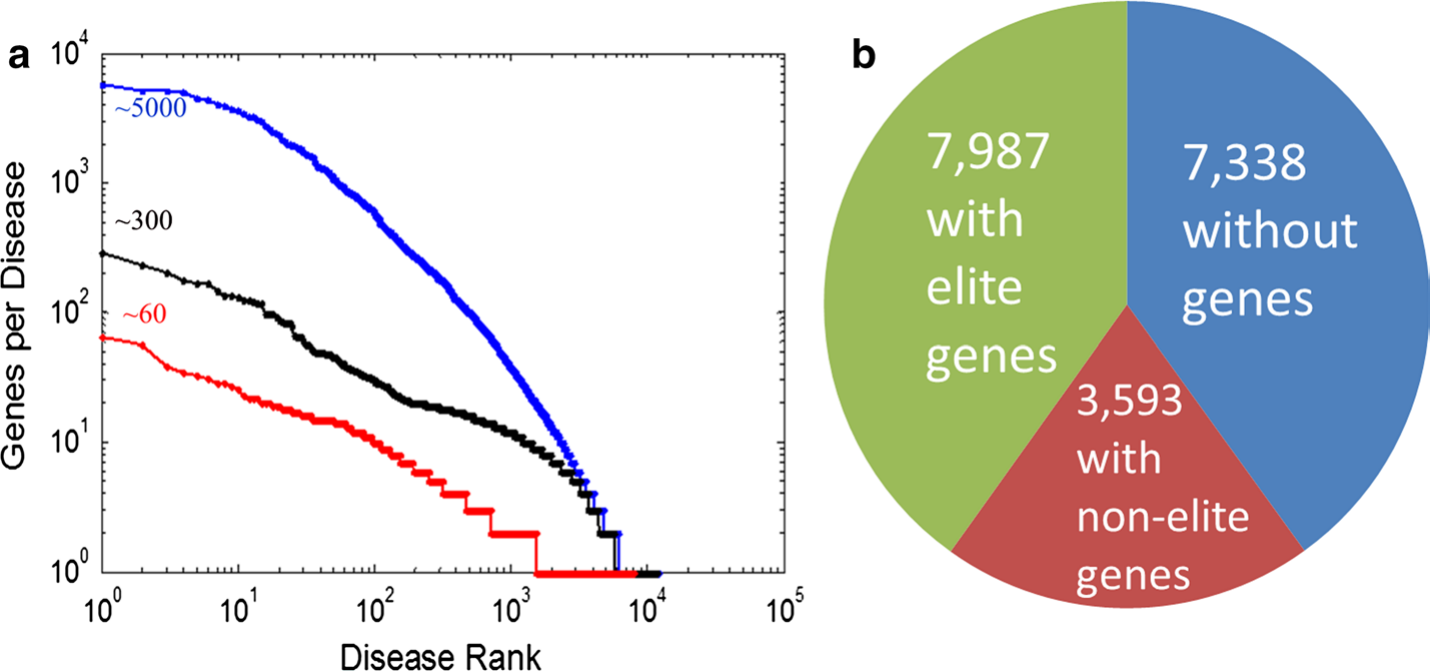
**a** Gene promiscuity filtering. Elite genes (*red*) connections via reliable sources such as OMIM, Orphanet, UniProtKB SwissProt and more. Text mining processes highlight additional associations. The number of genes associated with a disease before (*blue*), and after (*black*) filtering (see “Methods”) is shown. **b** Sources for gene information. About two-thirds of MalaCards diseases have associated genes. The data are based on MalaCards version 1.09

A conservative level of gene-disease connections is essential for enhancing the NGS interpretation specificity of VarElect. This prompted us to define three levels of association strength in the gene-disease network (Fig. 3). When considering all disease-gene relationships in our knowledge-base, i.e. all cases in which D-G >0, a very large network is obtained, connecting 22,280 genes to 11,580 disease entries, with some diseases having as many as 5000 associated genes. To provide more specific information, we applied filtration heuristics to reduce the number of gene disease connections (e.g. removing publications that provide more than 5 gene-disease associations, typically large scale studies which are more prone to providing noisy data). This reduced the connected gene count to 10,615, with no more than 300 genes per disease, and with nearly all diseases having 25 genes or fewer each. The number of gene-disease associations was reduced to 14.6%, involving 47% of the genes prior to filtration. Our gene-disease network is based on this connectivity level. Finally, it was deemed necessary to highlight gene-disease relations that are curated and evidence-based, for which reason we defined elite genes as the associations coming from curated sources such as OMIM and Orphanet. The elite gene-disease network has 4500 genes and 8000 diseases, with 99% of diseases having 10 or fewer genes (Fig. 3). The disease nodes of this reduced network are termed “elite diseases”, and its edges are likewise termed “elite edges”. An indication of the effectiveness of the data/text mining sources is that after the filtering step they still retain 75% of the elite edges in the network.

### The VarElect NGS interpretation tool—direct gene to phenotype associations

**Fig. 4:**
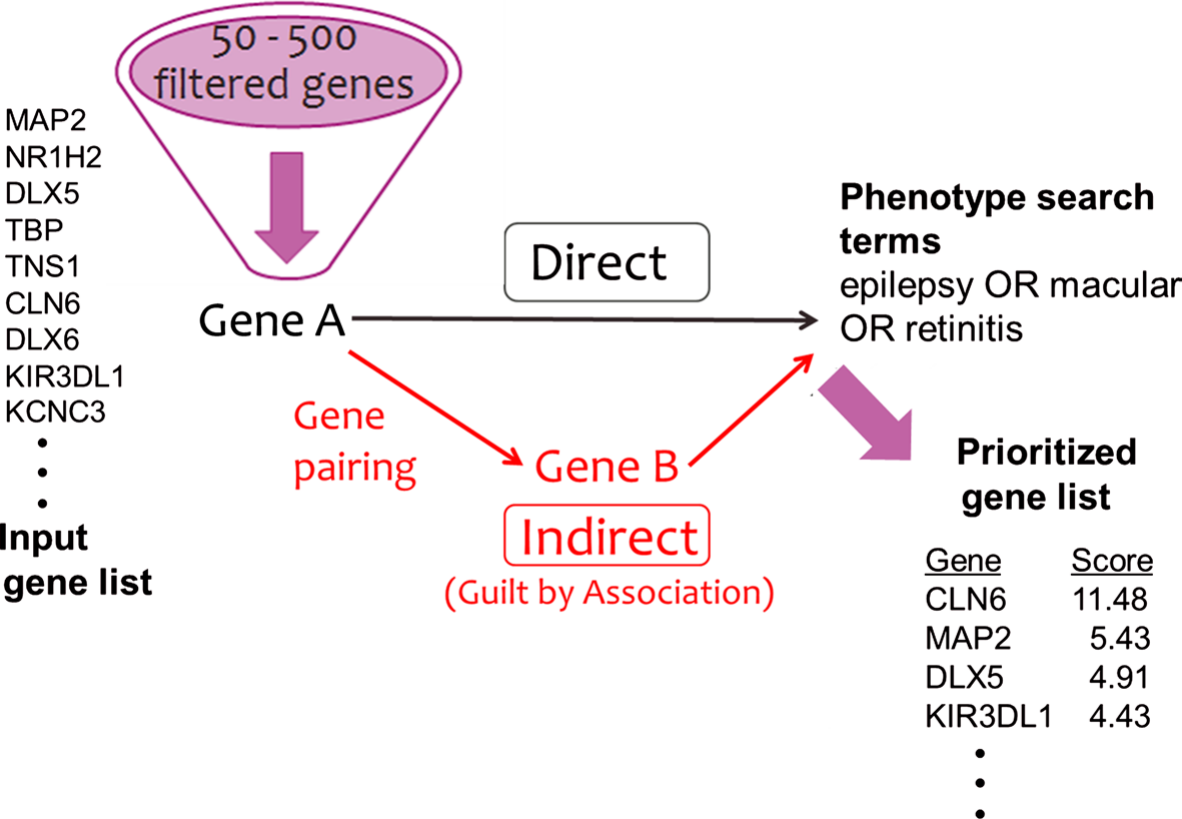
VarElect is used for prioritization of a filtered list of variation-harboring genes resulting from NGS experiments. A major strength of VarElect is its capacity to use implicating genes for creating indirect as well as direct connections between a gene and simple or complex phenotypes

NGS analyses identify non-reference variants in a specific subject. The total count of such variants ranges from tens of thousands for whole-exome sequencing to hundreds of thousands for whole-genome sequencing. Typically, only one or just a few variants are expected to be significant for the relevant disorder. In a filtering stage, parameters such as genetic model, rarity in the population, predicted protein impact, and gene evolutionary conservation help shorten the variants list to a few hundred variants, or even just a few dozen variants. Further focusing towards the identification of the causative disease genes requires NGS interpretation, i.e. seeking relationships between a variant-harboring gene and specific phenotype/disease terms. This is sometimes done manually, by consulting numerous heterogeneous databases. An alternative pipeline is to take advantage of a computerized knowledgebase. The GeneCards suite, including GeneCards, MalaCards and PathCards (the database of human biological pathways) [9] offers an effective way to do the latter, with a wealth of automatically-mined information, integration algorithms, as well as powerful unconstrained search and scoring capabilities linked to the VarElect NGS interpreter [11] (Fig. 4).

VarElect, as well as TGex—a broader VarElect-based NGS annotation and interpretation platform [11], infer direct and indirect links between genes, and diseases/phenotypes. The direct mode relies on the combined power of GeneCards and MalaCards, i.e. their shared gene-disease network, as well as their textual association capacities. The evidence for the obtained gene-phenotype relations is portrayed via the “MiniCards” mechanism originally instituted in the GeneCards search [5], and which displays the hit context within the card, with hyperlinks to the source databases. VarElect compares favorably with several popular NGS phenotyping tools [11], and it therefore provides an efficient way of ranking NGS-inferred genes by the strength of relatedness to disease keywords.

To date, VarElect has helped solve ~20 clinical cases in our own laboratory (e.g. [16–19]). In one such case, we studied a 6 year-old boy of Bukharian ancestry with atypical epilepsy combined with retinitis pigmentosa. Following whole-exome sequencing of the patient and his two healthy parents, we identified 81 rare homozygous variants in the patient, which were in a heterozygous state in both parents. We submitted this “medium-sized list” of 63 mapped genes along with the phenotype search terms; ‘epilepsy OR macular OR retinitis’ to VarElect. The resulting analysis placed CLN6 first with a long margin (Fig. 4). The patient had a homozygous missense variation (V148D) in this gene with zero population frequency, and protein impact genomic evolutionary rate profiling (GERP) score [20] of 5.14 (“highly damaging”). The mutation has not been previously reported in the context of this specific syndrome. Further functional studies are being performed to prove causality. Importantly, the result would not have materialized with GeneCards data or MalaCards data alone, underscoring the importance of their joint contribution to VarElect. Following this discovery, the patient was clinically diagnosed with accuracy, enabling appropriate genetic counselling and preimplantation diagnosis for the family in the event of future pregnancies.

### Indirectly-inferred connections in VarElect

In the indirect (or “guilt by association”) mode, VarElect can capitalize on the GeneCards suite’s varied gene-to-gene relationships to identify the relevance of NGS-derived genes that have no relationship to the entered phenotype terms on their own (Fig. 4). A major contribution to this comes from protein–protein interactions, as well as integrated pathway information from PathCards [8].

Pathway databases represent collections of genes and their interactions, mapped onto biological processes. Most of the information relevant to pathway definition comes from high-throughput protein–protein interactions [21–23], and from specific studies of cellular function having different emphasis and coverage [24]. There are significant inconsistencies caused by author preferences, and incompatibilities in data formats. Thus, pathways often provide an idiosyncratic view of biological mechanisms. PathCards was constructed to address such challenges, by aggregating 3215 pathways from 12 sources into 1073 SuperPaths, thus reducing redundancy, and maximizing informativeness [8].

VarElect’s indirect mode may further avail itself of the GenesLikeMe suite member (formerly GeneDecks Partner Hunter [13]). This tool relates genes to each other by shared attributes. These include sequence paralogy, GO terms, protein domains, mouse phenotypes, publications and tissue expression patterns. RNA expression patterns are derived from adapting GTEx data [25], and protein expression patterns stem from our human integrated protein expression database (HIPED), encompassing the integration of 4 proteomic data sources [26]. In VarElect, GenesLikeMe facilitates the generation of scored gene-to-gene matrices based on user-selected weighted combinations of attributes.

A good illustration of the strength of this approach is the case of a family diagnosed with systemic capillary leak syndrome. As previously reported [11], VarElect identified a promising candidate gene for this condition (*TLN1*), which was indirectly related to the phenotype through implicating genes associated with the phenotype.

### Disease-based pathways

**Fig. 5:**
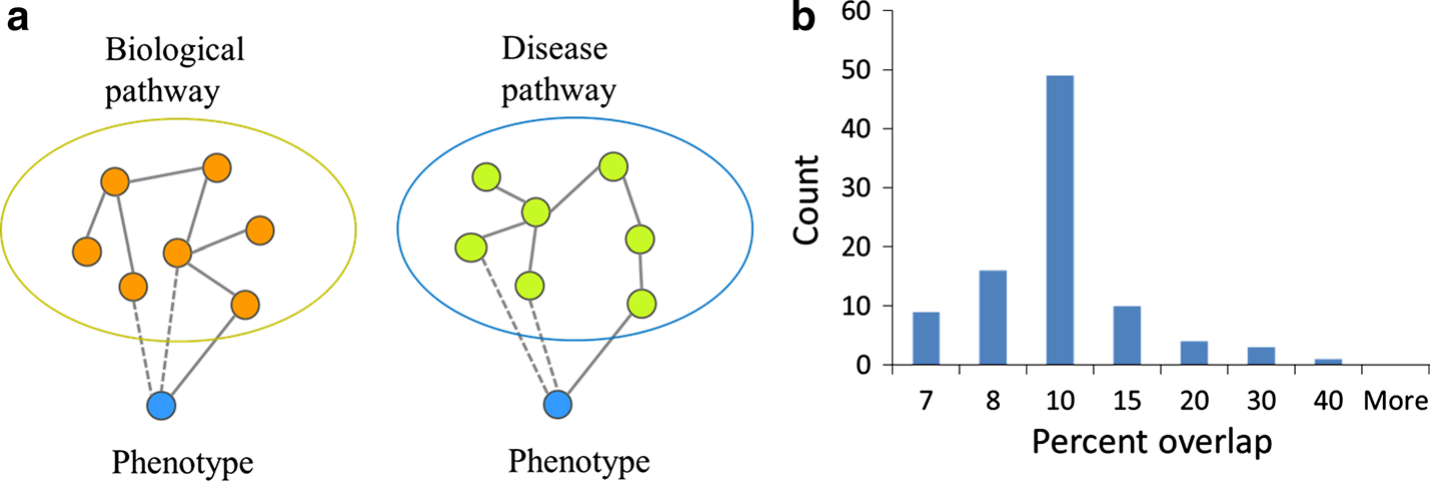
**a** Genes within a biological pathway allow for inference of disease relation to other pathway members (guilt by association). The same role can be played by genes associated with a disease, defined here as a “disease pathway”. **b** Genes within a disease pathway need not show significant overlap to canonical biological pathways. It was found that only 11.4 ± 5.5% of gene pairs in disease pathways also belonged to the same SuperPath. Therefore, disease pathways entail a new point of view for functional gene grouping

In PathCards, pathways are defined by biological insights regarding the functionalities of sets of genes. We note that PathCards regards each pathway as a “bag of genes” without regard to topological features such as “gene A activates gene B”. We have explored the use of this kind of simplified yet highly useful “compositional” view to delineate an alternative definition of pathway boundaries that reflect the disease-gene network. We propose to regard all of the genes for a given disease as a “disease pathway” (Fig. 5a). This is done by using the MalaCards integrated and filtered list of genes for each disease. While PathCards already includes a small number of pathways named after a disease, such as “Parkinson’s Disease Pathway”, our endeavour is much more comprehensive, defining thousands of novel pathway-like entities. We note that genes associated with a specific disease may be completely unrelated molecularly. In other words, genes belonging to a disease pathway seldom belong to the same standard biological pathways (Fig. 5b). Thus, in the realm of VarElect, a tool aimed at disease-gene relations, such newly inferred gene-to-gene relations may be beneficial for NGS disease interpretation. An example is as follows: In VarElect, if the search term is a disease name, all genes related to the disease in our disease-gene network will become hits. On the other hand, if one searches with a phenotype term, the indirect mode kicks into action. This shows not only the genes directly related to the term, but also all genes that reside within the same disease pathway shared with a gene associated with the phenotype (Fig. 4).

**Fig. 6:**
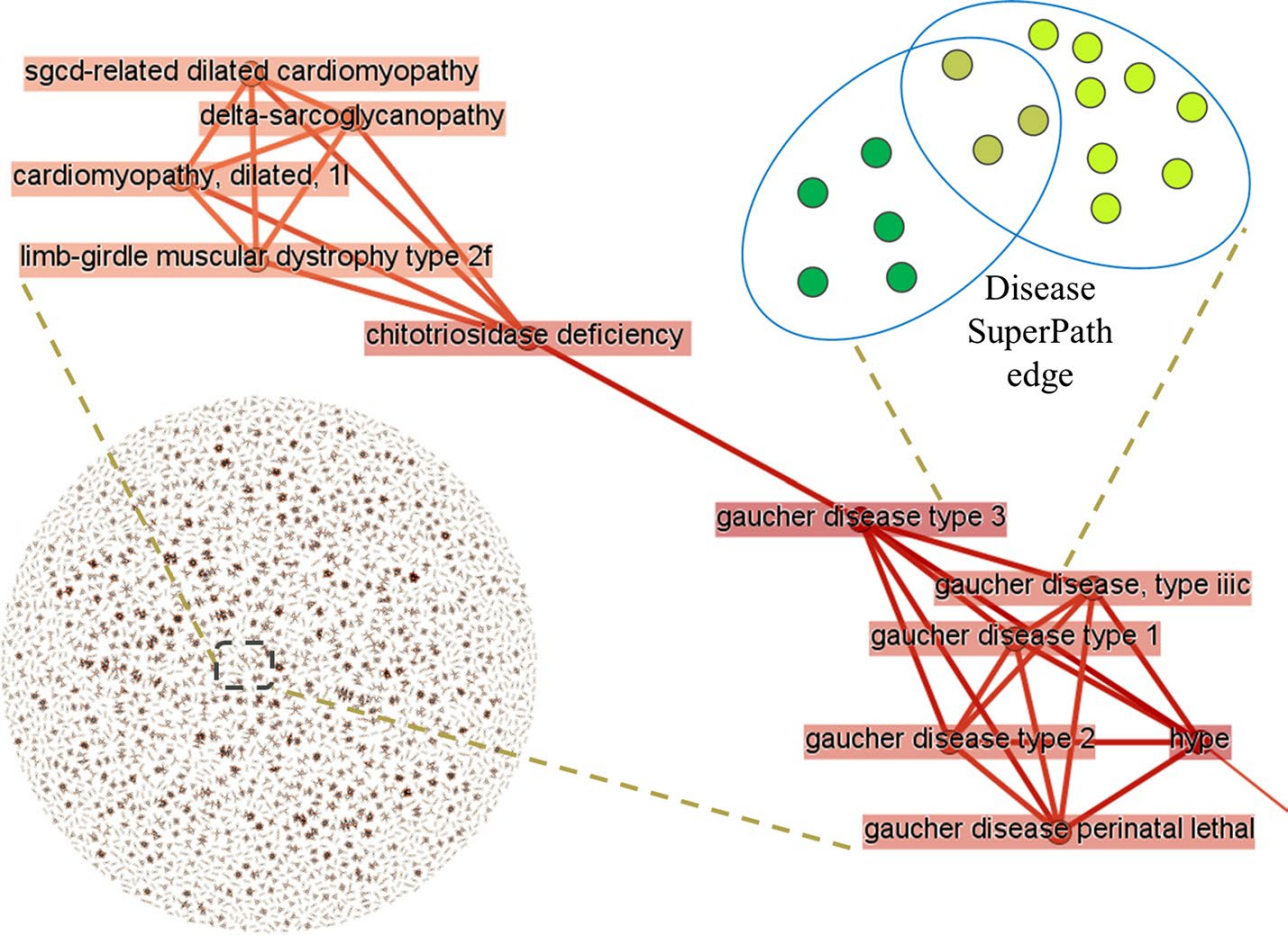
Disease SuperPaths are created by applying the PathCards biological pathway clustering algorithm to disease pathways, as explained in the text. *Bottom left—*the collection of all disease SuperPaths; *Center*—a specific disease SuperPath that appears to strongly represent Gaucher disease types, and diseases with related etiology.* Each rectangular edge* is a disease pathway, shown at the *top right* as an oval circumference, containing the disease genes that constitute the disease pathway. A connecting *red line* is an edge in the disease SuperPath, defined by gene sharing between the two disease pathways (genes shared by two ovals); The disease SuperPaths provide novel (indirect) disease–disease association, whereby two disease pathways that share no associated genes are linked to each other via the fact that there is a third disease pathway that shares gene with both (see text)

To further enhance the gene-to-gene relations gleaned through disease pathways, we used the PathCards algorithm [8] in order to coalesce disease pathways into disease SuperPaths (Fig. 6). The power of such action is in casting a wider net, whereby genes that do not share any individual disease pathway become related through the sharing of a disease SuperPath. Such a process may also be viewed as a means for augmenting the scope of disease relatedness (Fig. 1). Disease pathways become connected by mutual gene sharing (signifying an edge in a disease SuperPath), thus defining a collection of disease SuperPaths (Fig. 6 bottom left). This brings about a situation of indirect disease relations. This is exemplified in the specific case shown in Fig. 6 central part, whereby Gaucher disease type iiic and Gaucher disease type 3 have overlapping disease pathways (i.e. are related by gene sharing, Fig. 6 top right), signified by having a direct edge between them. The same is true for Gaucher disease type 3 and chitotriosidase deficiency. But significantly, Gaucher disease type iiic and chitotriosidase deficiency are only indirectly related (Fig. 6): they are 2 edges away, thus they share no associated genes. But they are linked via the fact that there is a third disease pathway (Gaucher disease type 3) that significantly shares genes with both.

### Preparing for whole-genome sequencing analyses

GeneCards encompasses a comprehensive collection of 101,976 ncRNA entries, integrated from 15 different data sources [3]. An integration algorithm is used, based on mapping to genomic coordinates via GeneLoc, another GeneCards suite tool [9]. VarElect can make use of this wealth of information to assist in identifying the involvement of ncRNA gene variations in disease.

Addressing transcription regulation, GeneCards is in the process of introducing regulatory element entries, with a special focus on the rather uncharted realm of distant-acting enhancers. This is based on the Ensembl regulatory build [27] as well as several other sources. GeneCards now displays a UCSC custom track showing all genes along with regulatory elements, so that users can inspect and judge relationships among them. In parallel, we are constructing a probability-based model for these associations, which will provide scores for regulatory element variations in relation to individual genes, allowing pipeline analyses by VarElect of variants residing outside the exome [28].

## Conclusions

GeneCards, MalaCards, and the other members of the GeneCards suite provide a rich resource for biomedical discovery within the gene-disease universe. Furthermore, with the envisioned addition of regulatory elements to the GeneCards database, connections between regulatory elements and diseases could also be established, thereby enriching the information regarding associations between diseases and genetic variation. VarElect, in conjunction with such databases and their connectivity, is an effective tool for supporting the interpretation experimental NGS analyses of disease. As demonstrated, VarElect is capable of rapidly generating both direct and indirect gene-disease connections, thereby facilitating new discoveries which would have otherwise been cumbersome to make. This is described in great detail in a recently published paper [11]. As exemplified, our databases and tools provide a rich infrastructure for large scale network analysis that could prompt promising drug targets and suggest alternative indications for existing drugs by examining the disease network interconnectivity. Along with preparedness for whole-genome sequencing, we believe that our databases and tools will provide an invaluable resource for researchers and clinicians, offering an effective capacity to investigate the molecular underpinning of diseases in the upcoming era of high throughput medical genomics.

## Methods

### Gene-disease network

As described earlier, two scores are computed for gene-disease associations: D-G is computed as a weighted sum of individual scores derived from 8 sources of information: OMIM, ClinVar, Orphanet, SwissProt-Humsavar, GeneTests, DISEASE, Novoseek and GeneCards [14, 15, 29]. The score values depend on the level of manual curation of the information source, and on the significance assigned by the source to its different annotation classes. For example, “molecular basis known” in OMIM (score of 500), “pathogenic mutation” in ClinVar (score of 400), “causative germ-line mutation” in Orphanet (score of 350), “causative variation” in Humsavar (score of 300) or appearance in GeneTests (score of 100) get considerably higher scores than “role in phenotype” or “Genetic linkage” in OMIM (score of 50). The last 3 sources in the above sources list, GeneCards, DISEASE and Novoseek, are based on data/text mining, and give rise to even lower source-specific scores as well. The GeneCards score is based on searching the disease name within the GeneCard (the specific card) of the relevant gene (excluding the disorders section). In MalaCards we define an elite gene for a disease as a gene with D-G >2.5. The inverse score, G-D, appears in GeneCards and indicates the importance of the genes with respect to the different diseases, giving a bonus to elite associations. Figure 2 displays the MalaCards elite gene disease network which groups related diseases, and thus highlights novel disease–disease associations, as well as novel gene-disease associations.

### Gene-disease promiscuity filtering

We employ heuristics for filtering the data in order to reduce spurious gene-disease associations per disease. This process is achieved by using a filtering threshold as determined by ${ \log }_{ 10} \left( {\text{Nm}} \right) = { \log }_{ 10} \left( {\text{Ne}} \right) + \left( {{ \log }_{ 10} \left( {\text{Nt}} \right) - { \log }_{ 10} \left( {\text{Ne}} \right)} \right)/ 2$, where Nm is the number of remaining genes; Nt is the total number of genes; Ne is the number of elite genes, for Ne ≥ Nt. Gene disease associations were mined through GeneCards via running Elasticsearch for an exact match with main name of the disease using a non-stemmed index.

### Exome sequencing and bioinformatics analysis

Whole-exome sequencing was performed using the SureSelect Human All Exon kit 37–50 Mb (Aligent Technologies, Santa Clara, CA). Samples were sequenced using the Illumina HiSeq 2000 platforms (Illumina, Inc. San Diego, CA). The resulting reads were aligned to the reference genome (GRCh37/hg19) using the Burrows-Wheeler Alignment (BWA-0.5.10). Polymerase chain reaction duplicates were removed using picard-tools-1.59 (http://picard.sourceforge.net). Genetic differences relative to the reference genome were called using UnifiedGenotyper of the Genome Analysis Toolkit (GATK-1.6–11). High quality SNVs were obtained using the following criteria: consensus score ≥20, SNP quality score ≥20, and reads supporting SNP ≥3. High quality indels were obtained using the following criteria: consensus score ≥20, indel quality score ≥50, ratio of (reads supporting variant)/(reads supporting reference): 0.2–5.0, and reads supporting indel ≥3. Annotation was performed using either SnpEff-3.3 (Ensembl 73 database), the SequenceVariantAnalyzer software (SVA), DNAnexus software (Palo Alto, CA, USA), and an in-house script using ANNOVAR80 and the GeneCards database annotation.

Only protein-altering variants (stop gain/loss, start loss, frameshift, missense, splice-site) were included. The dbNSFP database was used to access the functional prediction of non-synonymous SNPs. We primarily focused on genotypes absent in control data sets including the dbSNP138-142, the 1000 Genomes project, NHLBI GO Exome-sequencing project (http://evs.gs.washington.edu/EVS/), the ExAc browser http://exac.broadinstitute.org, 240 in-house controls of different Israeli ethnic origins and the internal control cohort comprised of 3027 subjects enrolled in the Center for Human Genome Variation (CHGV) through Duke institutional review board-approved protocols. Among all heterozygous variants only de novo or compound heterozygous variants were kept. The available protein predicting datasets such as PolyPhen2, SIFT, MutationTaster and LRT were used to predict mutations deleteriousness.

### The VarElect tool for NGS interpretation and clinical examples

The methodology used by the VarElect tool is described in [11]. The clinical example outlined in this paper included a 6 year-old boy of Bukharian ancestry with atypical epilepsy combined with retinitis pigmentosa (RP epilepsy). The patient’s syndrome also includes atonic seizures, abnormal EEG recordings, and hypopigmented macule. Following whole exome sequencing of the patient and his two healthy parents, we identified rare homozygous variants in the patient which were in a heterozygous state in both parents. The list of genes harboring these mutations was used in VarElect together with relevant keywords, to obtain a ranked list of candidate genes.

### Disease pathways

A disease pathway is defined by us as the set of genes associated in MalaCards with a given disease. For analysis of the overlap between disease pathways and biological pathways (Fig. 5), 92 randomly selected disease pathways sized 15–20 genes were analyzed against all 1073 SuperPaths in PathCards. All pair combinations within a disease pathway were tested against all pathways. The percentage pairs that belonged to the same SuperPath was calculated by the number of pairs that exist in the same biological pathway divided by the number of all possible pairs within the disease pathway.

For the creation of the disease pathway network (Fig. 6) we applied the PathCards algorithm to all sets of elite genes sets for all diseases. Clustering criteria for sets are applied according to the algorithm described in [8]. Visualization is done using Gephi [30].

## Abbreviations

GERP: genomic evolutionary rate profiling
GWAS: genome-wide association study
HIPED: human integrated protein expression database
ncRNA: non-coding RNA
NGS: next generation sequencing
OMIM: online mendelian inheritance in man
WGS: whole-genome sequencing
